# Probability and kinetics of rupture and electrofusion in giant unilamellar vesicles under various frequencies of direct current pulses

**DOI:** 10.1371/journal.pone.0304345

**Published:** 2024-06-10

**Authors:** Md. Tariqul Islam Bhuiyan, Mohammad Abu Sayem Karal, Urbi Shyamolima Orchi, Nazia Ahmed, Md. Moniruzzaman, Md. Kabir Ahamed, Md. Masum Billah

**Affiliations:** 1 Department of Physics, Bangladesh University of Engineering and Technology, Dhaka, Bangladesh; 2 Radiation, Transport and Waste Safety Division, Bangladesh Atomic Energy Regulatory Authority, Agargaon, Dhaka, Bangladesh; 3 Department of Physics, Jashore University of Science and Technology, Jashore, Bangladesh; University of Tennessee, UNITED STATES

## Abstract

Irreversible electroporation induces permanent permeabilization of lipid membranes of vesicles, resulting in vesicle rupture upon the application of a pulsed electric field. Electrofusion is a phenomenon wherein neighboring vesicles can be induced to fuse by exposing them to a pulsed electric field. We focus how the frequency of direct current (DC) pulses of electric field impacts rupture and electrofusion in cell-sized giant unilamellar vesicles (GUVs) prepared in a physiological buffer. The average time, probability, and kinetics of rupture and electrofusion in GUVs have been explored at frequency 500, 800, 1050, and 1250 Hz. The average time of rupture of many ‘single GUVs’ decreases with the increase in frequency, whereas electrofusion shows the opposite trend. At 500 Hz, the rupture probability stands at 0.45 ± 0.02, while the electrofusion probability is 0.71 ± 0.01. However, at 1250 Hz, the rupture probability increases to 0.69 ± 0.03, whereas the electrofusion probability decreases to 0.46 ± 0.03. Furthermore, when considering kinetics, at 500 Hz, the rate constant of rupture is (0.8 ± 0.1)×10^−2^ s^-1^, and the rate constant of fusion is (2.4 ± 0.1)×10^−2^ s^-1^. In contrast, at 1250 Hz, the rate constant of rupture is (2.3 ± 0.8)×10^−2^ s^-1^, and the rate constant of electrofusion is (1.0 ± 0.1)×10^−2^ s^-1^. These results are discussed by considering the electrical model of the lipid bilayer and the energy barrier of a prepore.

## 1. Introduction

Biomembranes exhibit distinct electrical properties that can be effectively manipulated through the application of electric field. At weak direct current (DC) pulses of electric field, the membrane can deform under the influence of electric stresses [1]. At strong pulses, transient pores form in the membranes (i.e., electroporation), which dramatically increases the membrane permeability. Electroporation technology is utilized for the transmembrane transport of drugs, gene, proteins, and other molecules in the fields of medicine, food processing, and specific environmental contexts [2–5]. In the irreversible electroporation (IRE) technique, a series of high electric field (in V/cm) DC pulses are applied to ablate cancer cells/tumors and rupture vesicles in a non-thermal process [6, 7]. A related phenomenon of electroporation called electrofusion can be employed to merge two cells or vesicles that are in close proximity, enabling the creation of hybrid cell-cell, vesicle-vesicle, or cell-vesicle fusion products [8, 9]. In a clinical study, it was observed that elevating the frequency of AC field exerts an inhibitory effect on the growth of cancer cells (e.g., HeLa), attributed to the alteration of membrane potential at distinct frequencies [10]. The effect of frequency of the AC field on the distribution of electrical stresses on the cell surface has been investigated and found that frequency has a significant effect on cell deformation and membrane permeability [11]. Very recently, the dynamics of cell death (e.g., Chinese hamster ovary cells (CHO), mouse melanoma cells (B16F1), and rat heart myoblast cells (H9c2)) have been observed by changes in metabolic activity and membrane integrity at high electroporation intensities [12]. This suggests that cell death resulting from electroporation is not an immediate all-or-nothing response but rather a dynamic process that occurs over an extended period of time.

Most of the electroporation and electrofusion in lipid vesicles have been performed by varying the electric field of DC and some cases AC with a constant frequency [8, 13, 14]. Previously, we have investigated the IRE in giant unilamellar vesicles (GUVs) by varying the surface charge and salt concentration in a physiological buffer [15], cholesterol concentration in the lipid membranes [16], concentration gradient between the exterior and interior of GUVs (e.g., osmotic effect), and sugar concentration [17, 18]. In addition, the electrotension (electric field induces electrotension) dependent kinetics of vesicle rupture has been investigated in which the rate constant of rupture formation increases with the applied tension [19]. Basically, the probability of rupture and kinetics of rupture are two important parameters found from the statistical analysis of many ‘single GUVs’ which helps to understand the elementary process of pore formation in the lipid bilayer due to the electric and mechanical tension [20–23]. The theoretical fitting to the experimental data on the tension (electric or mechanical) dependent rate constant of pore formation gives the opportunity to find the most significant intrinsic parameter of the lipid membrane called pore edge tension, which is responsible for closing the pore [22, 24–26]. Moreover, the direct evidence of transient pores was visualized in the bilayer [27] and quantified the potential energy barrier associated with lipid bilayer electroporation [28, 29].

Although there are several reports on the DC pulses induced poration and fusion in GUVs, almost all the investigations are performed by changing the electric field with a single frequency. Moreover, the probability and the kinetics of electroporation and electrofusion is rarely investigated under various frequencies of DC pulses. As the vesicle/cell membranes behave as a parallel resistor-capacitor circuit, it is indispensable to investigate the responses of GUVs under a range of frequencies. Through the investigation of the impacts of frequency on GUVs, the optimum frequency that is effective in either electroporation or facilitating the electrofusion has been obtained. The results of several independent experiments comprising many ‘single GUVs’ are analyzed statistically for determining the average time, probability, and the rate constant of rupture and electrofusion.

## 2. Materials and methods

### 2.1 Chemicals and reagents

1,2-dioleoyl-*sn*-glycero-3-phospho-(1′-*rac*-glycerol) (sodium salt) (DOPG) and 1, 2-dioleoyl-*sn*-glycero-3-phosphocholine (DOPC) were purchased from Avanti Polar Lipids Inc. (Alabaster, AL). 1,4-Piperazinediethanesulfonic acid (PIPES), bovine serum albumin (BSA), O,O´-Bis (2-aminoethyl) ethyleneglycol-*N*,*N*,*N´*,*N´*-tetraacetic acid (EGTA), sodium chloride, glucose, and sucrose were purchased from Sigma-Aldrich (Germany).

### 2.2 Preparation of GUVs

**Fig 1:**
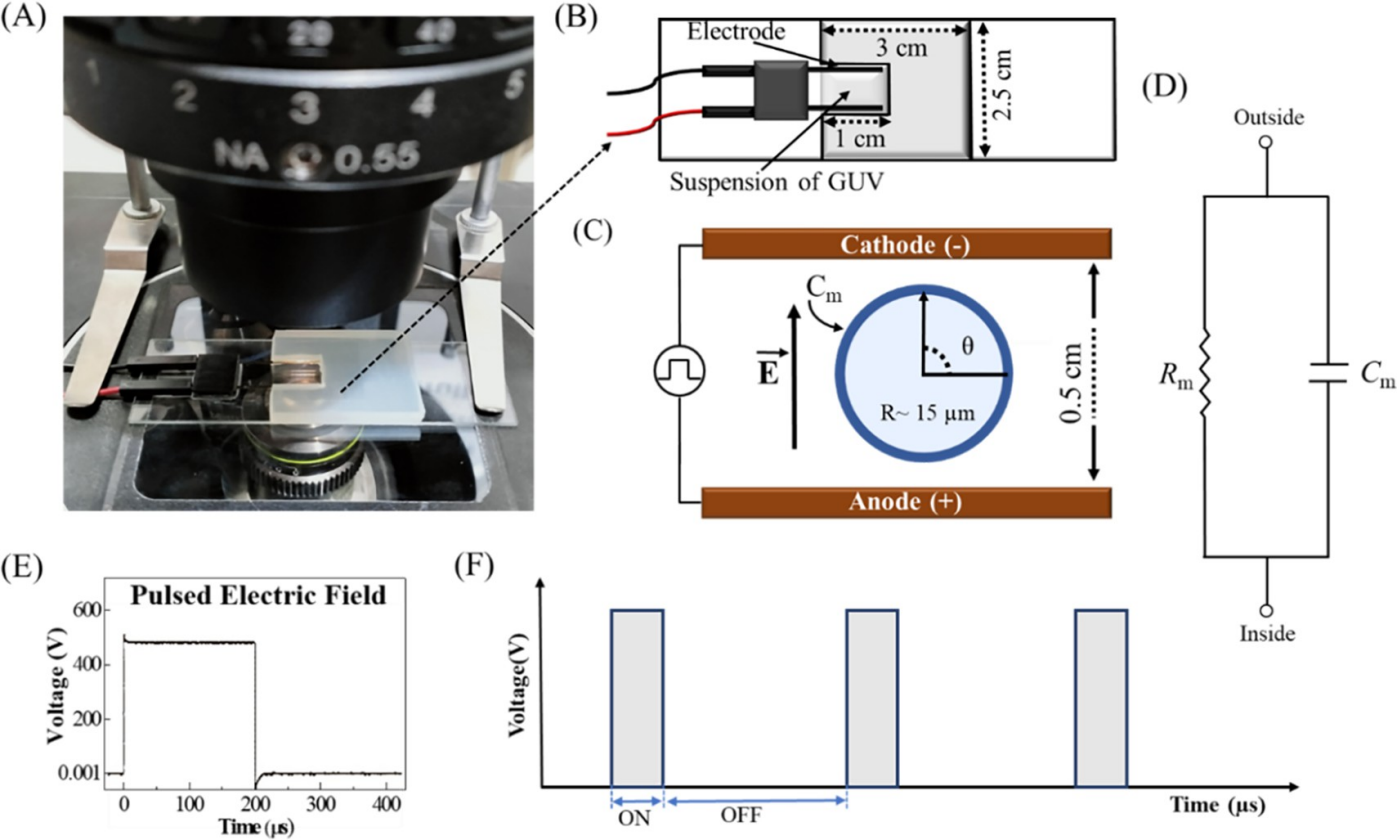
Experimental setup to apply the electric field on the GUVs. (A) Experimental set up of a microchamber at the stage of an inverted phase contrast microscope. The microchamber comprised a glass slide, a silicon rubber spacer, and a cover slip, forming a U-shaped structure. (B) Schematic representation of the U-shaped microchamber contained GUVs suspension and gold-coated electrode with proper configuration. (C) Schematic of a ‘single GUV’ exposed to the DC pulses of electric field. The direction of electric field (*E*) is shown in the figure. (D) The equivalent circuit of the lipid membrane, containing a resistor (*R*_m_) and a capacitor (*C*_m_) in parallel. (E) A DC pulse of electric field. (F) Illustration of DC pulses (rectangular pulses) with ON time (i.e., 200 μs) and OFF time (variable of time, i.e., 1800, 1050, 752, and 600 μs) along the x-axis. The y-axis indicates the applied electric field. The frequencies of the DC pulses were 500, 800, 1050, and 1250 Hz.

DOPG and DOPC lipids were used to prepare the DOPG/DOPC (40/60)-GUVs [here 40/60 indicates the molar ratio] by the natural swelling method [30] in a physiological buffer (10 mM PIPES, 150 mM NaCl, pH 7.0, 1mM EGTA). At first a mixture of 1 mM DOPG and DOPC of total amount 200 μL was taken into a glass vial and dried with a gentle flow of nitrogen gas for producing a thin, homogeneous lipid film. Then the vial was placed in a vacuum desiccator for 12 h for removing the remaining chloroform. The lipid film was pre-hydrated for 8 min at 45°C by adding an amount of 20 μL MilliQ water into the glass vial. After that the sample was incubated for 3 h at 37°C with 1 mL buffer containing 0.10 M sucrose for producing the suspension of GUVs. To separate the suspension of GUVs from lipid aggregates and multilamellar vesicles, the samples were subjected to centrifugation using 13000×*g* (here *g* is the acceleration due to gravity) at 20°C for 20 min using a centrifuge machine (NF 800R Centrifuge, Nuve, Turkey). Subsequently, the supernatant of the suspension was purified using the membrane filtering method in which the purified GUVs contained 0.10 M glucose in buffer as an external solution [31, 32]. An amount of 300 μL purified suspension of GUVs was transferred into a U-shaped hand-made microchamber (see Fig 1B) which was coated with 0.10% (w/v) BSA, dissolved in 0.10 M glucose containing buffer. An inverted phase contrast microscope (Olympus IX-73, Japan) with a 20× objective (numerical aperture: 0.45) was used to observe the GUVs at 25 ± 1°C (Tokai Hit Thermo Plate, Japan). The recording of GUVs images was done using a charged coupled camera of 25 frames per second (fps) (Model: DP22, Olympus) connected to the microscope.

### 2.3 Experimental setup for applying electric field in the microchamber interior

We have investigated the effects of frequency of DC pulses of electric field on a ‘single GUV’. A ‘single GUV’ refers specifically to the targeted GUV used for investigation upon the application of an external electric field. A field of view of raw image of GUVs at different time points is provided in the supporting information (S) (see S1). In addition, the ‘single GUV’ method [20] is described briefly for understanding the statistical analysis of the experimental data in the S2. The experimental setup of a microchamber at the stage of an inverted phase contrast microscope is depicted in Fig 1(A). The microchamber consists of a glass slide, a silicon rubber spacer, and a cover slip, arranged to create a U-shaped structure. The size of the microchamber and electrode configuration are shown in Fig 1(B). Fig 1(C) shows an illustration of a ‘single GUV’ between the two gold coated electrodes. Usually, vesicle suspension contains different sizes of GUVs. The electrical model of a membrane is characterized by a capacitor *C*_m_ and a resistor *R*_m_ as shown in Fig 1(D). A rectangular DC pulse is used in the investigations (Fig 1E). The detailed experimental technique has been reported previously [33]. Fig 1(F) shows an illustration of the rectangular DC pulses of electric field with fixed ON time of 200 μs and varying OFF times of 1800, 1050, 752, and 600 μs, generates corresponding frequency 500, 800, 1050, and 1250 Hz.

### 2.4 Rupture and electrofusion of GUVs

At first, we explain how DC pulses of electric field induces lateral electric tension in the membranes of GUVs. Let us consider a spherical ‘single GUV’ of radius *R* (Fig 1C). The electric field propels the inside and outside charged ions towards the vesicle membrane, causing the membrane to become charged, similar to the behavior of a capacitor. The accumulation of charges along the membrane generates transmembrane voltage (*V*_m_) according to the Schwan’s equation [34]:

$$V_{m}=1.5RE\;\cos\;\theta (1-e^{-t/\tau })$$ (1)

where, the characteristic charging time of the membrane is *τ*, the angle, *θ*, between the direction of the field *E* and the normal to the bilayer surface vary from 0 to 90°. *V*_m_ = 1.5*RE* = *V*_c_ at *θ* = 90°, which is the ‘critical membrane voltage for breakdown of GUV [35]. Generally, if *R* = 16 μm and *E* = 332 V/cm, *V*_m_ = 0.8 V. The transmembrane voltage leads to induce the lateral electric tension *σ*_e_ in the membrane as follows [35]:

$$\sigma_{e}=\varepsilon_{m}\varepsilon_{0}\left(\frac{h}{2h_{e}^{2}}\right)V_{m}^{2}$$ (2)

where, *ε*_m_ is relative membrane permittivity (~4.5), *ε*_0_ is the permittivity of free space, *h* is the thickness of membrane (~4 nm) and *h*_e_ is the membrane dielectric thickness (~2.8 nm) [13, 36, 37]. After simplification, the following equation can be obtained for a spherical shaped GUV [19]:

$$\sigma_{e}=22.86\;R^{2}E^{2}\;[mN/m]$$ (3)

For simplicity, we considered uniform tension in the membrane induced by electric stress, although recent numerical results show non-uniform membrane tension for non-spherical (i.e., elliptical) shaped vesicles with different conductivity ratios between the inside and outside of the vesicle [38].

We examined a ‘single GUV’ in a microchamber. The size range of GUVs was 26−30 μm. At first, we measured the radius of each selected ‘single GUV’, and then determined the required electric tension, i.e., *σ*_e_ = 5.2 mN/m using Eq (3) for a specific frequency (say 500 Hz) by changing *E*. To induce the required membrane tension (*σ*_e_) to rupture a ‘single GUV’ or to fuse multiple GUVs of typical diameter, the corresponding range of electric field (*E*) was 320–340 V/cm to monitor the stochastic phenomena of the GUVs for a maximum time of 60 s. The time of rupture is defined as the moment when a ‘single GUV’ initiates the formation of rupture. Similarly, the time of electrofusion is defined as the moment when two or multiple GUVs commence the process of fusing into a single GUV. In both cases, the initial time point (*t* = 0) is defined as the moment when electric tension is just applied to the membranes. The recorded video was employed to identify the initiation of rupture or fusion formation. We conducted 3 to 4 independent experiments (e.g., *N* = 3–4), examining 13 to 16 GUVs (e.g., *n* = 13–16) in each individual experiment, for each specified frequency. In the case of electrofusion of ‘two-connected GUVs’ or ‘multiple-connected GUVs’, we measured the radius of bigger size GUV among the connected GUVs and then calculated *σ*_e_ = 5.2 mN/m using Eq (3). By statistically analyzing the data, we determined both the probability and rate constant of rupture and electrofusion. As larger GUV requires higher membrane tension to induce pore formation, we chose a larger size GUV for the electrofusion. It is noteworthy that we examine a single event such as rupture or electrofusion in each microchamber. Due to technical constraints, individual GUVs were examined for a maximum duration of 60 s. GUVs that did not undergo rupture/fusion within this 60 s timeframe were classified as intact GUVs for a particular chamber. We have calculated the average time given the distribution of rupture/fusion event that have occurred within 60 s.

## 3. Results

We initially present the qualitative description of the experimental results on the rupture and electrofusion of GUVs at different frequencies of direct current (DC) pulses of electric field. Then the results are analyzed statistically for the quantification of different parameters, e.g., average time, probability, and rate constant of rupture and electrofusion.

### 3.1 Rupture of DOPG/DOPC (40/60)-GUVs under different frequencies

**Fig 2:**
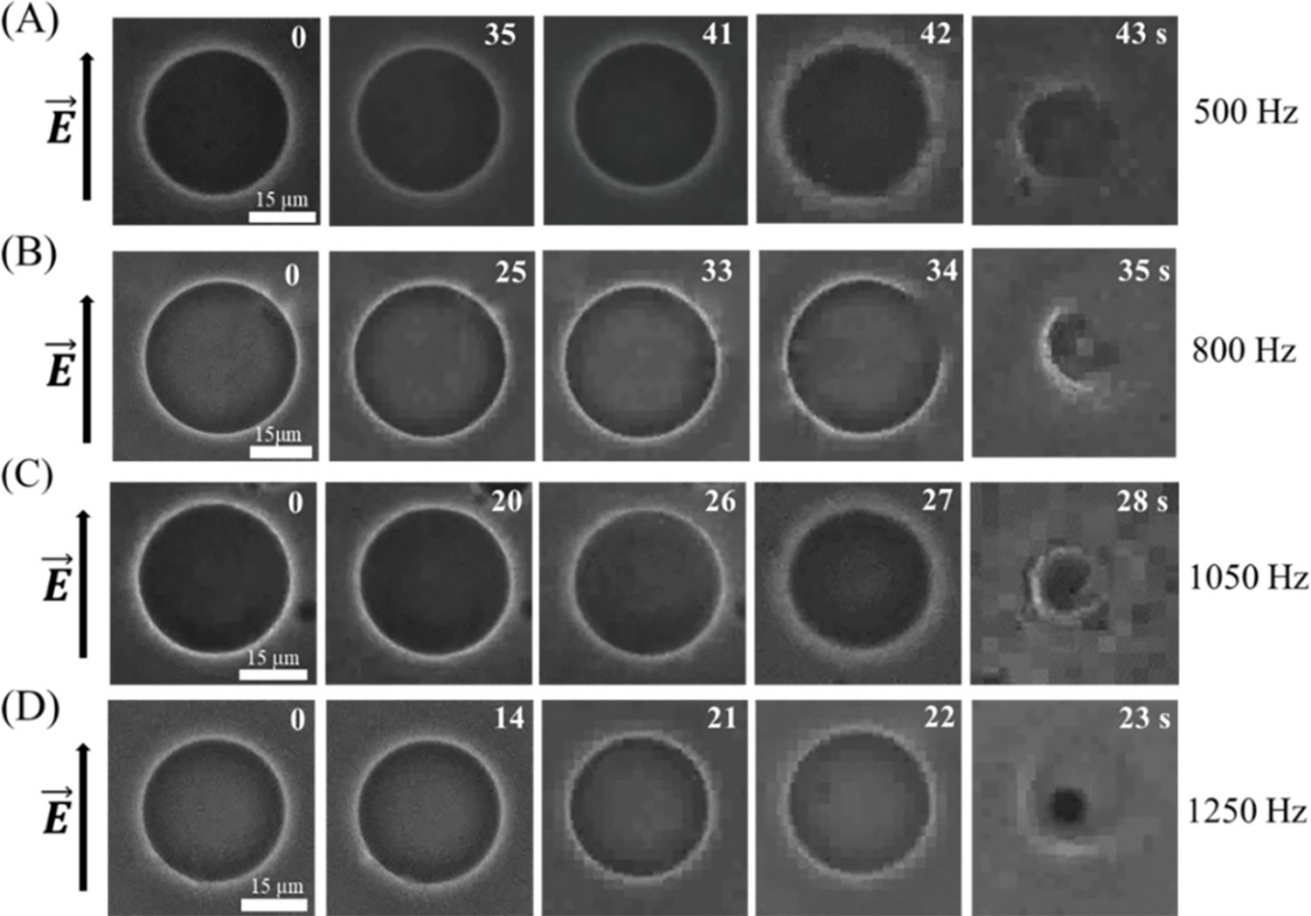
Rupture of DOPG/DOPC (40/60)-GUVs at different frequencies of DC pulses under *σ*_e_ = 5.2 mN/m. The phase contrast images of the rupture of GUVs at (A) 500, (B) 800, (C) 1050, and (D) 1250 Hz. The electric field (*E*) direction is shown with an arrow on the left side of each figure. The numbers in each image show the time in seconds after applying *E*.

We investigated the rupture in DOPC/DOPG (40/60)-GUVs at different frequencies. A representative result of one independent experiment is presented in Fig 2. At first, we consider the case of 500 Hz. Before applying the tension, *σ*_e_ = 5.2 mN/m in the membranes, the ‘single GUV’ remains perfectly spherical in shape at 0 s as shown in Fig 2(A). After applying the tension, the GUV remains intact with the same shape at observed at 0 s until time 41 s. At time 42 s, the shape of the GUV becomes distorted and started to rupture (i.e., rupture occurs). The structure of the GUV vanishes at time 43 s (Fig 2A). We have performed the same experiment and obtained the similar phenomena of GUVs at 800, 1050, and 1250 Hz. The corresponding times for rupture of GUVs are observed at 34 s, 27 s, and 22 s. Through optical microscopy, the nano-sized pores were not directly visible at the time of rupture, but there was a noticeable decrease in the contrast of the GUVs. In certain instances, the observation of internal content leakage was noted. The initiation of electropore formation in the lipid bilayer is detected within a few nano to microseconds [39]. Therefore, the utilization of a high-resolution camera, along with other instrumental setups, is necessary. In this paper, our focus is on rupture formation, which can be readily observed through optical microscopy. It is well accepted that when the nanopores underwent rapid enlargement over time, GUVs experienced complete rupture while the electric tension persisted. This process is akin to the aspiration of a ‘single GUV’ using the micropipette technique [25].

**Fig 3:**
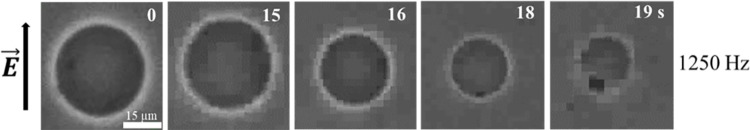
Size change before rupture of DOPG/DOPC (40/60)-GUV at 1250 Hz of DC pulses under *σ*_e_ = 5.2 mN/m. The phase contrast images of the progressively decrease of GUV and resulting rupture of GUV. The electric field (*E*) direction is shown with an arrow on the left side of each figure. The numbers in each image show the time in seconds after applying *E*.

In some cases, at 1250 Hz, we have observed the change in size of GUVs before rupture. A representative result of a ‘single GUV’ is shown in Fig 3. Before applying the tension at time *t* = 0 s, the size of GUV was 30 μm. After applying the tension, the size remains same until 5 s. At time 16 s, the size reduces to 20.7 μm, and at 18 s it becomes to 17.7 μm. The GUV becomes complete rupture at 19 s. The size reduction can be attributed to the expel of lipids from the bilayer due to the submicron pore formation at 16 s in the membranes [40, 41].

**Fig 4:**
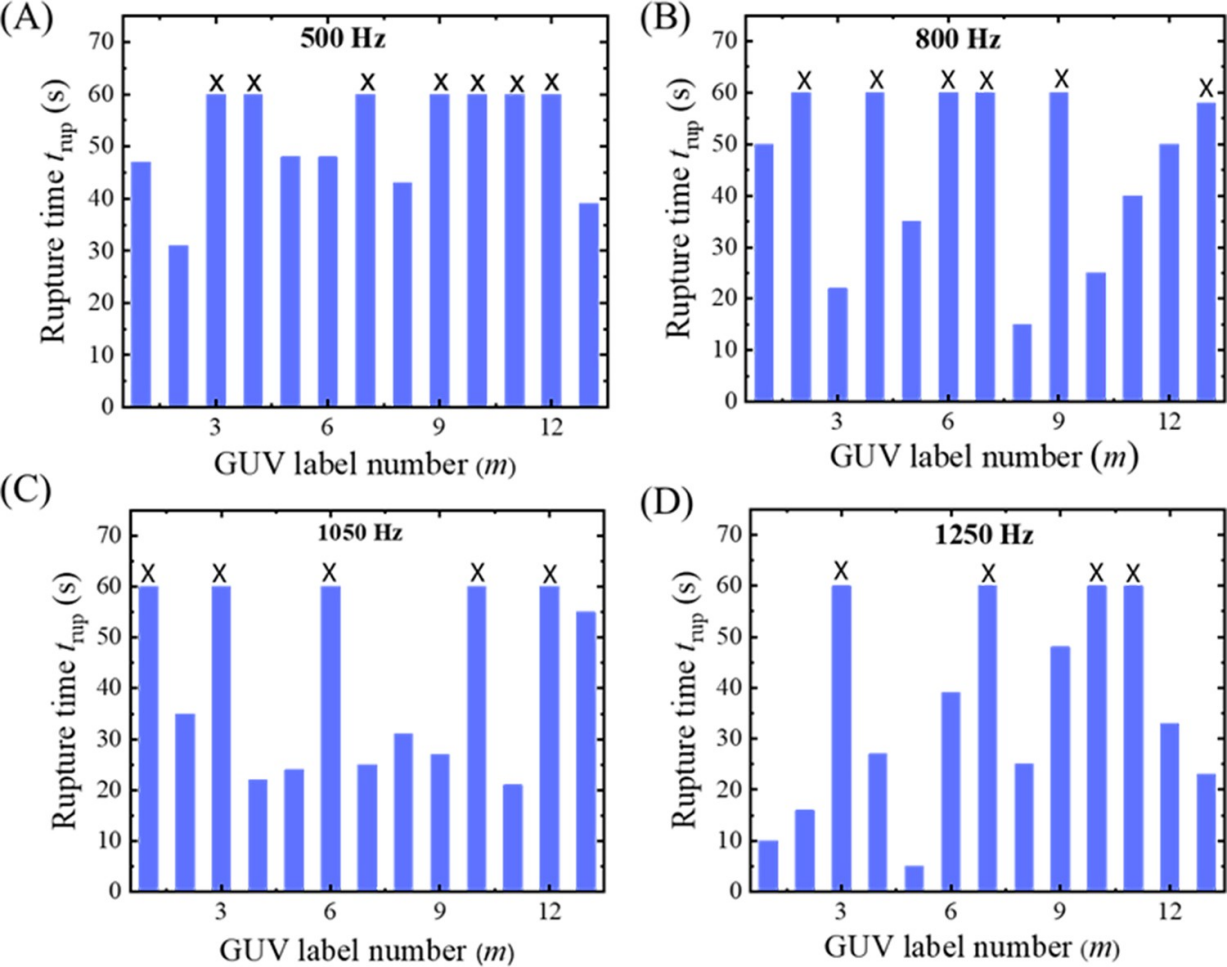
Rupture time (*t*_rup_) of DOPG/DOPC (40/60)-GUVs at different frequencies of DC pulses under *σ*_e_ = 5.2 mN/m. Stochastic rupture of several ‘single GUVs’ in one independent experiment at (A) 500, (B) 800, (C) 1050, and (D) 1250 Hz. The x-axis shows the GUV label number (*m*). The cross mark (×) above the bars indicates the intact GUVs until time 60 s.

Next, we have investigated the rupture of several ‘single GUVs’ in each frequency. In Fig 4(A), the first, second, and fifth GUVs were ruptured at 47, 30, and 48 s, respectively. The third and fourth GUVs were not ruptured within 60 s. At 500 Hz, among the 13 GUVs observed, there were 6 instances of GUV rupture (as depicted in Fig 4A). Additionally, at 800, 1050, and 1250 Hz, the numbers of ruptured GUVs were 7, 8, and 9, respectively (as presented in Fig 4B–4D, respectively). Thus, the time of rupture of several ‘single GUVs’ at a specified frequency is not same showing a stochastic nature as reported in the literature [22, 42, 43]. This is clearly indicating that the number of ruptured GUVs becomes higher at higher frequencies. The rupture time (on average) decreases as the frequency increases. The quantitative analysis of stochastic ruptures of several ‘single GUVs’ is considered in 3.3.

### 3.2 Electrofusion in two ‘single DOPG/DOPC (40/60)- GUVs’ under different frequencies

**Fig 5:**
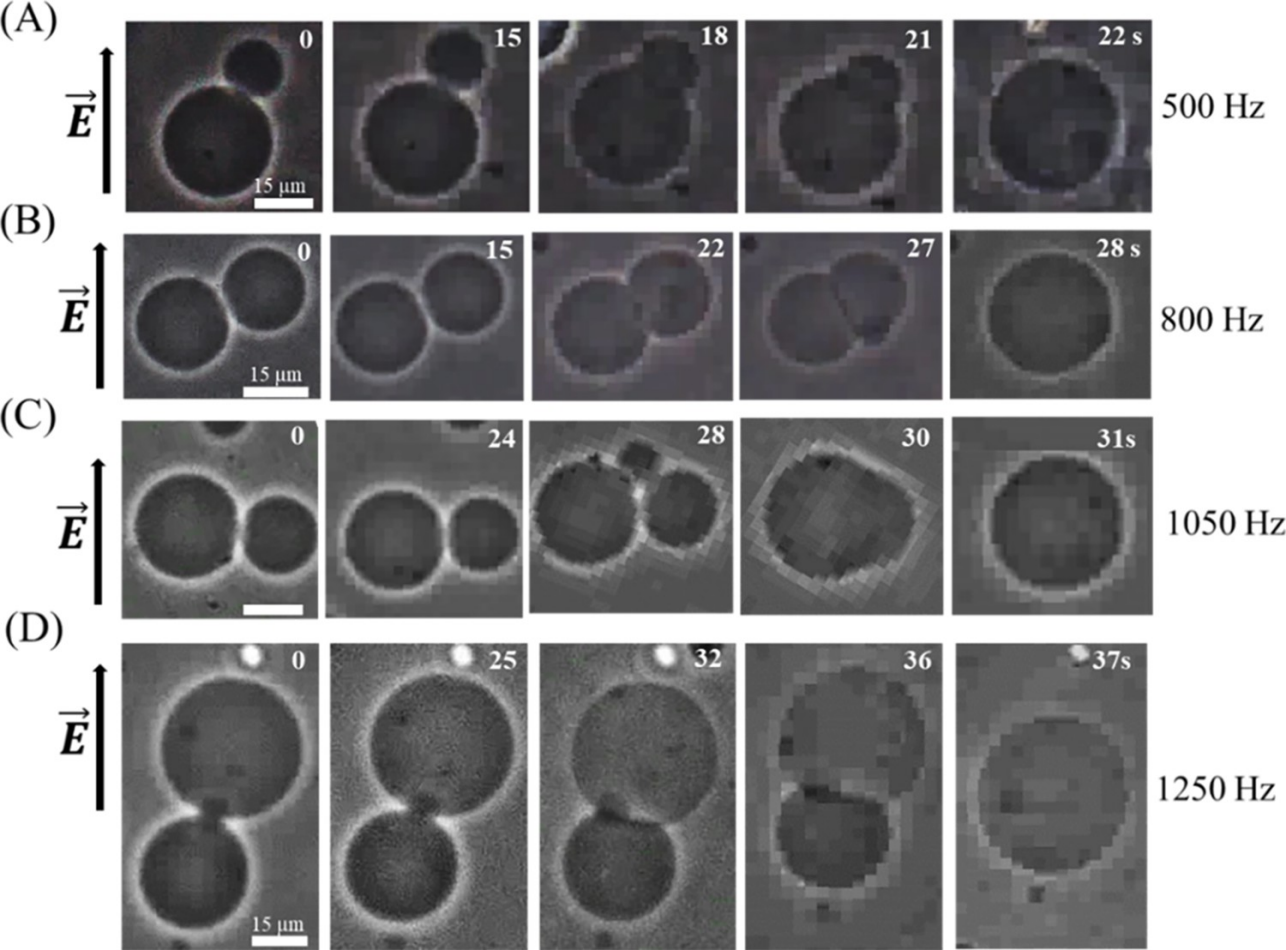
Fusion of DOPG/DOPC (40/60)-GUVs at different frequencies of DC electric pulses unser *σ*_e_ = 5.2 mN/m. The phase contrast images of the fusion of ‘two-connected GUVs’ at (A) 500, (B) 800, (C) 1050, and (D) 1250 Hz. The electric field (*E*) direction is shown with an arrow on the left side of each figure. The numbers in each image show the time in seconds after applying *E*.

In this section, we have presented the results of electrofusion of ‘two-connected GUVs’ at different frequencies under *σ*_e_ = 5.2 mN/m. A representative result of electrofusion of two GUVs is presented in Fig 5 for several frequencies. At first, we consider the case of 500 Hz. Before applying the tension, the two connected GUVs remain perfectly spherical in shape at 0 s as shown in Fig 5(A). In the presence of tension, the GUVs remain intact with the same shape and size at observed at 0 s until time 15 s. In this stage the fusion neck is almost zero (see Fig 11 for clear understanding of fusion neck). At time 18 s, the connected GUVs merge slightly (i.e., fusion neck moderate) and at time 21 s the GUVs merges a greater degree (i.e., fusion neck enlarged), and the GUVs merged completely at 22 s (Fig 5A). Thus, the ‘two-connected GUVs’ transformed to a single GUV. It means the two GUVs looks like a dumbbell shaped single GUV at 21 s and this dumbbell shape disappeared within ~ 1 s and converted to a spherical shaped GUV. The electrofusion of GUVs is observed at 21 s for 500 Hz (Fig 5A), 27 s for 800 Hz (Fig 5B), 30 s for 1050 Hz (Fig 5C), and 36 s for 1250 Hz (Fig 5D). It is assumed that the electrofusion occurs due to the simultaneous pore formation in the membranes at contact point [44]. The phenomena of GUVs become similar for several independent experiments.

**Fig 6:**
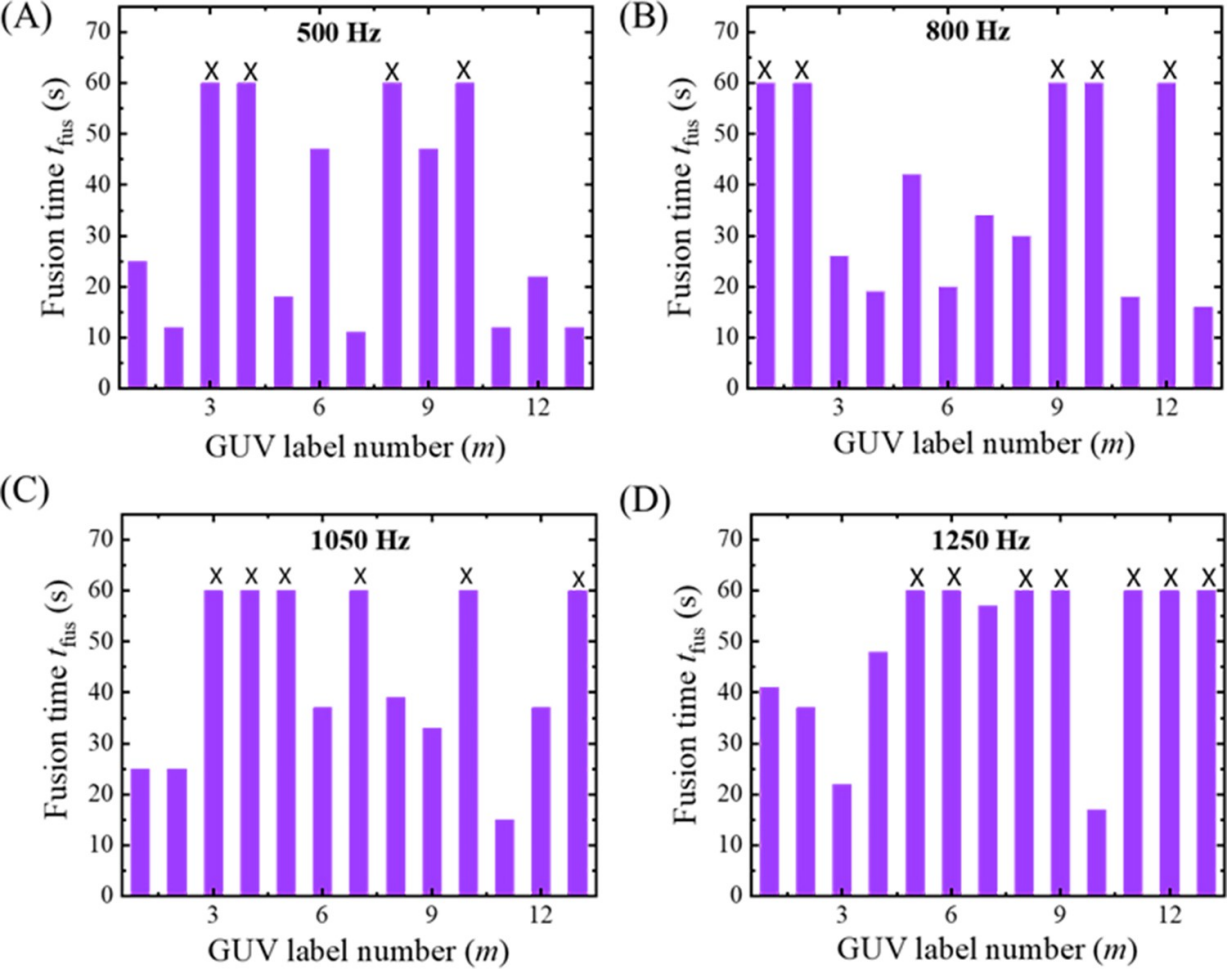
Electrofusion time (*t*_fus_) of DOPG/DOPC (40/60)-GUVs at different frequencies of DC pulses under *σ*_e_ = 5.2 mN/m. Stochastic fusion of several ‘two-connected GUVs’ in one independent experiment at (A) 500, (B) 800, (C) 1050, and (D) 1250 Hz. The x-axis shows the GUV label number (*m*). The cross mark (×) above the bars indicates the intact GUVs until time 60 s.

Next, we have investigated the electrofusion of several ‘two connected GUVs’. Here, we have presented the results of one independent experiment comprising 13 ‘two connected GUVs’ in each frequency. A number of 9 GUVs are fused out of 13 GUVs at 500 Hz (Fig 6A). The same procedure was applied in several ‘two-connected GUVs’ for 800, 1050, and 1250 Hz, and observed the stochastic fusion nature of vesicles [22, 42, 43]. Out of 13 GUVs, the numbers of fused GUVs were 8 for 800 Hz (Fig 6B), 7 for 1050 Hz (Fig 6C), and 6 for 1250 Hz (Fig 6D). The experimental results shown in Fig 6 indicate that the number of fused GUVs reduces following an increase in frequency. The quantitative analysis of electrofusion is presented in 3.3.

### 3.3 Frequency dependent probability of rupture and electrofusion in DOPG/DOPC (40/60)-GUVs

**Fig 7:**
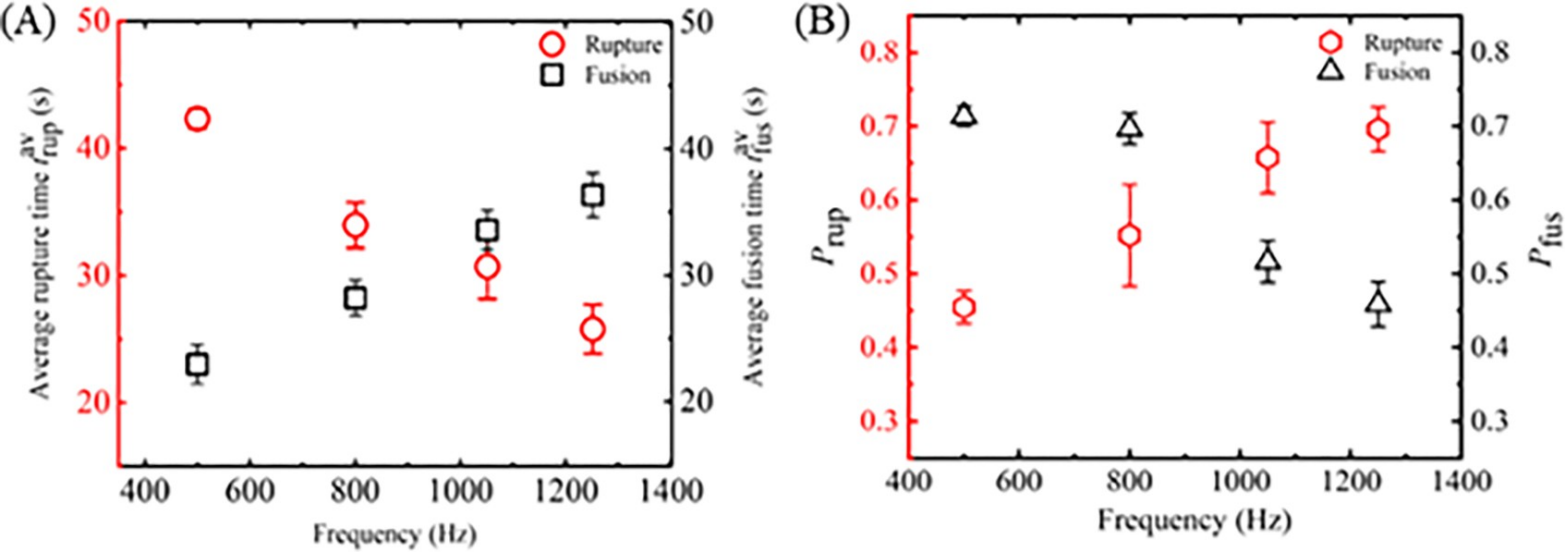
Frequency dependent average rupture time, average fusion time, rupture probability, and fusion probability of DOPG/DOPC (40/60)-GUVs under *σ*_e_ = 5.2 mN/m. Average values and standard deviations of each data were determined from several independent experiments.

In this section, the frequency dependent rupture and the electrofusion are analyzed statistically. Firstly, we have calculated the quantitative data of average time of rupture and electrofusion and also the probability of rupture and electrofusion. Fig 7(A) shows that the average rupture time decreases while average fusion time increases with increasing frequency. The average rupture time decreases 39.2% whereas the electrofusion time increases 37.08% for increasing the frequency from 500 to 1250 Hz. The tendency of decreasing the average time for rupture and the increasing the average time for electrofusion follows almost a linear function with frequency. Now the results of the probability of rupture (*P*_rup_) and probability of electrofusion (*P*_fus_) are presented. Fig 7(B) shows that the probability of rupture increases while probability of fusion decreases with increasing frequency. The probability of rupture increases 53.3% whereas the probability of electrofusion decreases 35.2% for increasing the frequency from 500 to 1250 Hz. Hence, the frequency enhances the possibility of electroporation whereas the opposite trend is shown for electrofusion. The data on the frequency dependent average time of rupture, average time of electrofusion, probability of rupture, and probability of electrofusion is shown in Table 1.

**Table 1:** Frequency dependent average time, probability, and rate constant of rupture and electrofusion of DOPG/DOPG (40/60)-GUVs.

Frequency *f* (Hz)	Average time of rupture $t_{rup}^{av}$ (s)	Probability of rupture *P*_rup_	Rate constant of rupture *k*_p_rup_ (s^-1^)	Average time of fusion $t_{fus}^{av}$ (s)	Probability of electrofusion *P*_fus_	Rate constant of electrofusion *k*_p_fus_ (s^-1^)
500	42.3 ± 3.5	0.45 ± 0.02	(0.8 ± 0.1)×10^−2^	22.9 ± 1.5	0.71 ± 0.01	(2.4 ± 0.1)×10^−2^
800	33.9 ± 1.8	0.53 ± 0.07	(1.3 ± 0.1)×10^−2^	28.2 ± 1.3	0.69 ± 0.02	(2.3 ± 0.4)×10^−2^
1050	30.6 ± 2.5	0.65 ± 0.05	(1.7 ± 0.5)×10^−2^	33.5 ± 1.6	0.52 ± 0.03	(1.2 ± 0.1)×10^−2^
1250	25.7 ± 1.9	0.69 ± 0.03	(2.3 ± 0.8)×10^−2^	36.4 ± 1.7	0.46 ± 0.03	(1.0 ± 0.1)×10^−2^

### 3.3 Rate constant of rupture and electrofusion in DOPG/DOPC (40/60)- GUVs under different frequencies

Now, the kinetics such as the rate constant of rupture (*k*_p_rup_) and rate constant of electrofusion (*k*_p_fus_) of the GUVs have been considered. The rate constant governing the rupture/fusion of GUVs stands as a pivotal parameter that elucidates the pore formation/electrofusion process within a collection of GUVs, subject to external influences. Precise insight into the kinetics governing the rupture of GUVs holds significant importance in comprehending the underlying mechanism driving the rupture/fusion phenomenon. To explain the stochastic phenomenon of rupture/electrofusion quantitatively, we considered a parameter *P*_int_ (*t*), meaning the probability of GUVs being in intact state at the instant of time, *t*, among all the examined GUVs. The value of *P*_int_ (*t*) considering rupture/electrofusion events as the transition from intact state to ruptured/fused state, is given by Eq (4) [19, 25, 45],

$$P_{int}(t)=exp(-k_{p}t)$$ (4)

where, *k*_p_ is the rate constant of rupture/fusion of GUVs and *t* is the duration of time to apply the tension in GUVs. We used *P*_int_rup_ for rupture and *P*_int_fus_ for electrofusion as for convenience.

**Fig 8:**
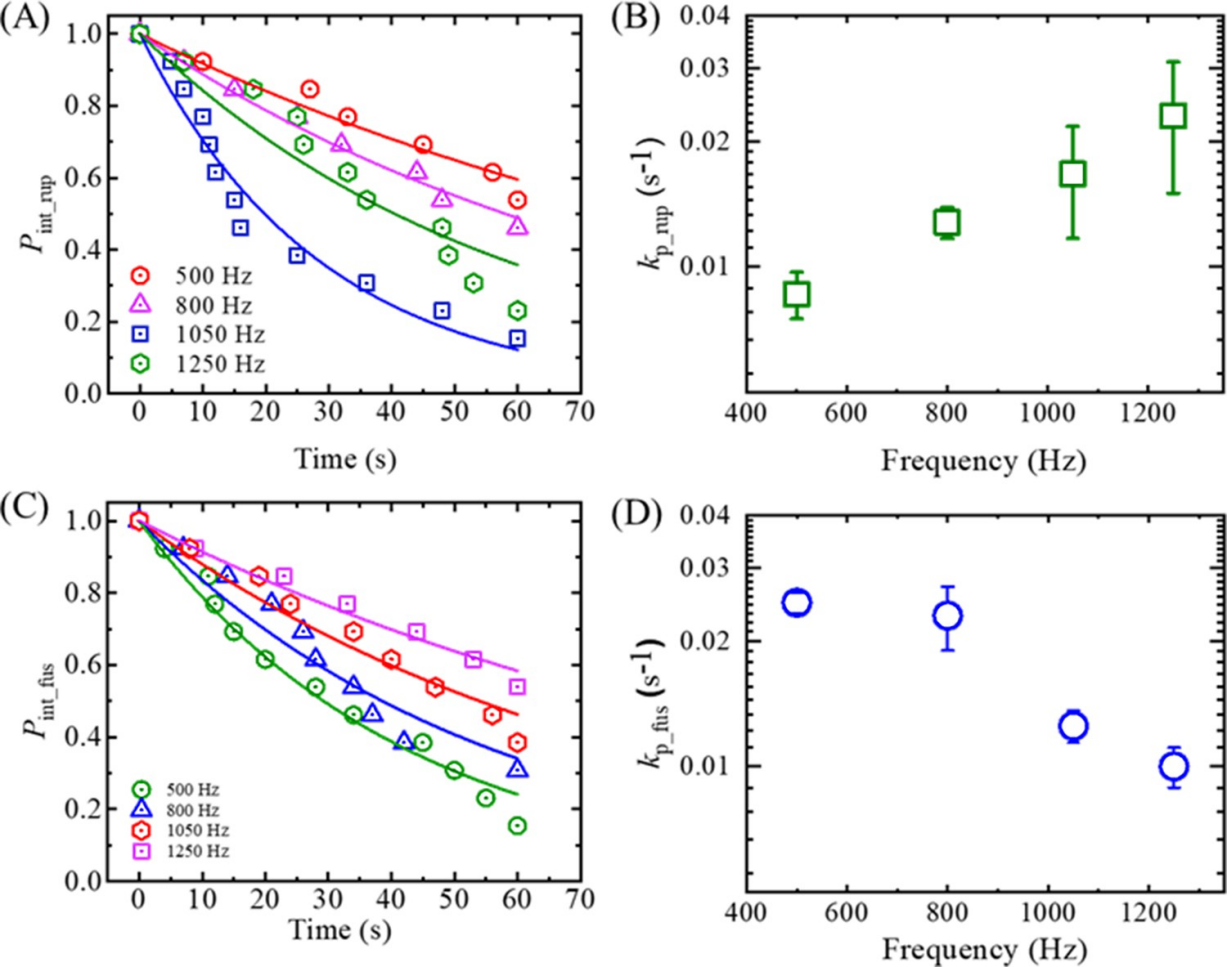
The time course of the fraction of intact DOPG/DOPC (40/60)-GUVs and the rate constant of rupture and electrofusion at different frequencies of DC pulses under *σ*_e_ = 5.2 mN/m. (A) The time course of the fraction of intact GUVs at 500, 800, 1050, and 1250 Hz in the case of rupture of vesicles. (B) The frequency-dependent rate constant of rupture of GUVs. (C) The time course of the fraction of intact GUVs at 500, 800, 1050, and 1250 Hz in the case of electrofusion of vesicles. (D) The frequency-dependent rate constant of electrofusion of ‘two-connected GUVs’. The experimental data in (A) and (C) is fitted using a single exponential decay function of Eq 4. Average values and standard deviations of each data in (C) and (D) were determined from several independent experiments comprising many ‘single GUVs’.

To determine the rate constant of rupture/electrofusion, the values of *P*_int_ (*t*) have been determined from the experiment data. It can be defined as *P*_int_ (*t*) = 1- *P*_rup_ or *P*_int_ (*t*) = 1- *P*_fus_ [46, 47]. Fig 8(A) shows the time course of *P*_int_rup_ (*t*) for different frequencies. It shows that the decrement of experimental data for *P*_int_rup_ versus time is a factor when applied frequency changes from 500 to 1250 Hz. The decrement is smaller at 500 Hz and higher at 1250 Hz. The experimental data on the *P*_int_rup_ versus time graph is well fitted by a single exponential decay function of Eq (4). From the fitted curves, the values of *k*_p_rup_ are obtained 0.8×10^−2^, 1.3×10^−2^, 1.7×10^−2^, and 2.3×10^−2^ s^-1^ for 500, 800, 1050 and 1200 Hz, respectively. The same experiments were carried out for several independent experiment (*N* = 3−4). The values of average *k*_p_rup_ are obtained (0.8 ± 0.1)×10^−2^, (1.3 ± 0.1)×10^−2^, (1.7 ± 0.5)×10^−2^, and (2.3 ± 0.8)×10^−2^ s^-1^ for 500, 800, 1050 and 1200 Hz, respectively, at 5.2 mN/m. Hence, the *k*_p_rup_ increases with frequency (Fig 8B). The result for 1250 Hz in Fig 8(A) represents an independent experiment, and the data exhibits slight fluctuation. However, the average value of *k*_p___rup_ (± SD) indicates an increasing trend with frequency. Next, we calculated the *k*_p_fus_ as described above. Fig 8(C) shows the time course of *P*_int_fus_ (*t*) for different frequencies. It shows that the decrement of experimental data for *P*_int_fus_ versus time is a factor when applied frequency changes from 500 to 1250 Hz. The decrement is faster at 500 Hz and slower at 1250 Hz. The experimental data on the *P*_int_fus_ versus time graph is well fitted by a single-exponential decay function of Eq (4). From the fitted curves, the values of *k*_p_fus_ are obtained 2.4×10^−2^, 2.3×10^−2^, 1.2×10^−2^, and 1.0×10^−2^ s^-1^ for 500, 800, 1050, and 1250 Hz, respectively. The same experiments were carried out for several times (*N* = 3−4) and the values of average *k*_p_fus_ were obtained (2.4 ± 0.1)×10^−2^, (2.3 ± 0.4)×10^−2^, (1.2 ± 0.1)×10^−2^, and (1.0 ± 0.1)×10^−2^ s^-1^ for 500, 800, 1050 and 1200 Hz, respectively, at 5.2 mN/m. Hence, the *k*_p_fus_ decreases with frequency (Fig 8D). The data on the frequency dependent rate constant of rupture and electrofusion is shown in Table 1.

As the time course of the fraction of intact GUVs (see Fig 8) follows a single exponential decay function, it confirms the stochastic nature of rupture/fusion events. Essentially, the fraction of intact GUVs has been derived from the probability of rupture/fusion of GUVs. Exponential decay results directly from events occurring randomly over time, where there exists a meaningful average rate for such occurrences.

### 3.4 Electrofusion in multiple DOPG/DOPC (40/60)- GUVs at 500 Hz

**Fig 9:**
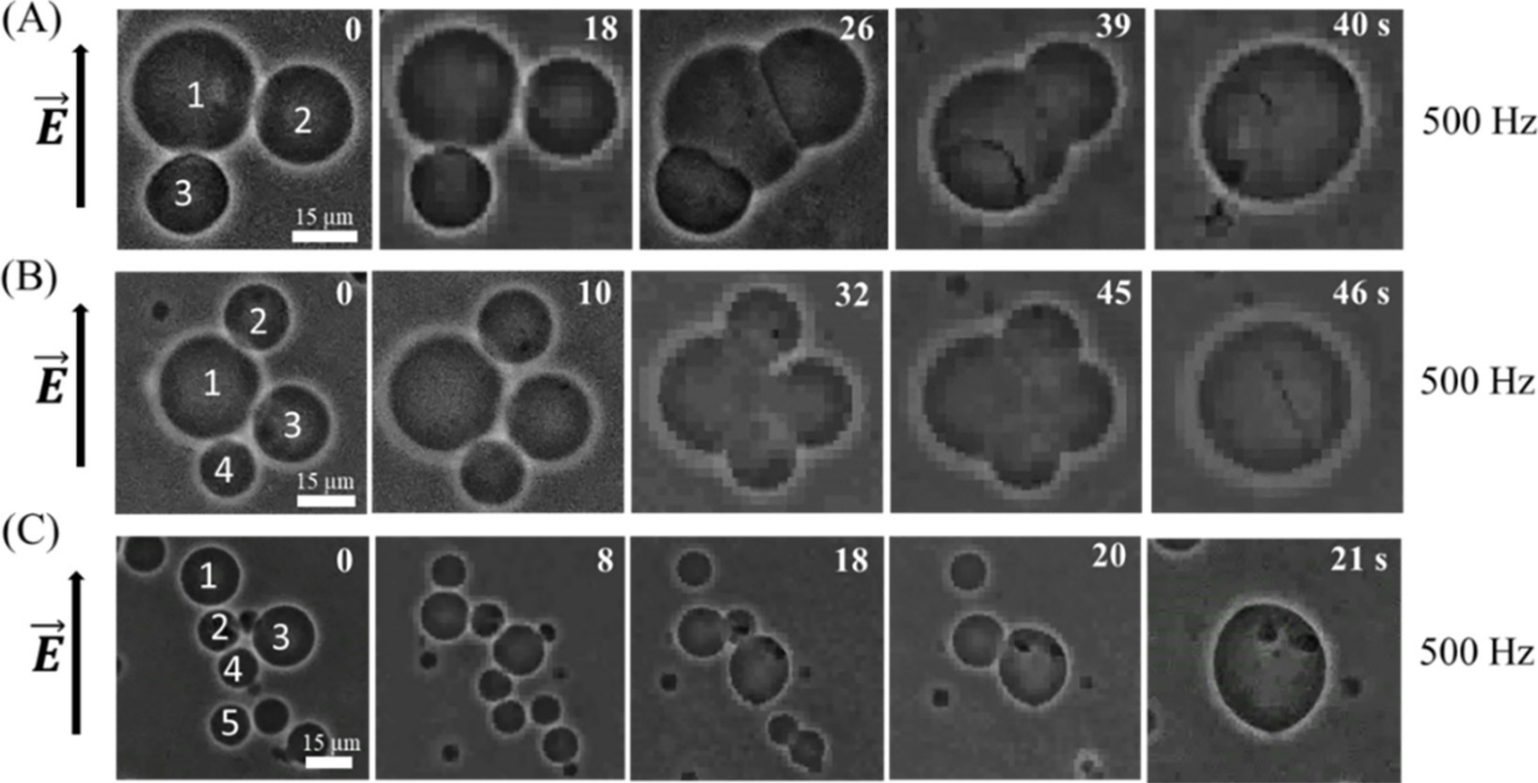
Electrofusion of multiple DOPG/DOPC (40/60)-GUVs at 500 Hz of DC pulses under *σ*_e_ = 5.2 mN/m. The phase contrast images of electrofusion of (A) ‘three-connected GUVs’, (B) ‘four-connected GUVs’, and (C) ‘five-connected GUVs’. The electric field (*E*) direction is shown with an arrow on the left side of each figure. The numbers in each image show the time in seconds after applying *E*.

In 3.2, we have described the results of electrofusion of ‘two-connected GUVs’ at different frequencies under *σ*_e_ = 5.2 mN/m. Here we focus the electrofusion of ‘multiple-connected GUVs’ at a fixed frequency, e.g., 500 Hz. A representative result of electrofusion of ‘three-connected GUVs’, ‘four-connected GUVs’, and ‘five-connected GUVs’ is shown in Fig 9. The phenomena to fuse the multiple GUVs is similar to that obtained for fusing of two GUVs as described in 3.2. The similar behavior of electrofusion is also observed for other frequencies. We have summarized the volume of each ‘single GUV’ as presented in Figs 5(A) and 9(A–9C) at 0 s (initial stage) and compared with the volume of fused GUV at final stage. Actually, we have considered to verify the equation, $\sum_{i=1}^{N_{t}}\frac{4}{3}\pi r_{i}^{3}=\frac{4}{3}\pi R^{3}$, where *r*_i_ is the radius of GUVs at 0 s (the GUVs contributing to electrofusion), *R* is the radius of GUV at final stage (the fused GUV), and *N*_t_ is the number of electro-fused GUVs. In Fig 5(A) at 0 s, *r*_1_ = 15.48 μm, *r*_2_ = 8.14 μm, and thus volume *V*_1_ = 4π/3 (*r*_1_^3^ + *r*_2_^3^) = 17811 μm^3^. At final stage (i.e., 46 s), volume *V*_2_ = 4π/3 (16.24)^3^ = 17951 μm^3^. Hence, *V*_2_/*V*_1_ = 1.008. We have calculated *V*_2_/*V*_1_ for the case of GUVs shown in Fig 9(A)–9C, and found the values as 1.002, 1.004, and 1.002, respectively. Thus, the average value with SD as *V*_2_/*V*_1_ = 1.004 ± 0.003 ≈ 1.0.

## 4. Discussion

In this research, we have investigated the rupture and electrofusion of cell-mimetic GUVs under various frequencies of DC pulses. Both the phenomena depend on the applied frequency. As the frequency increases the probability of rupture and the rate constant of rupture increases, whereas electrofusion shows the opposite nature. The mechanism of these phenomena is discussed in detail in 4.1 and 4.2.

### 4.1 Vesicle rupture and its mechanism

**Fig 10:**
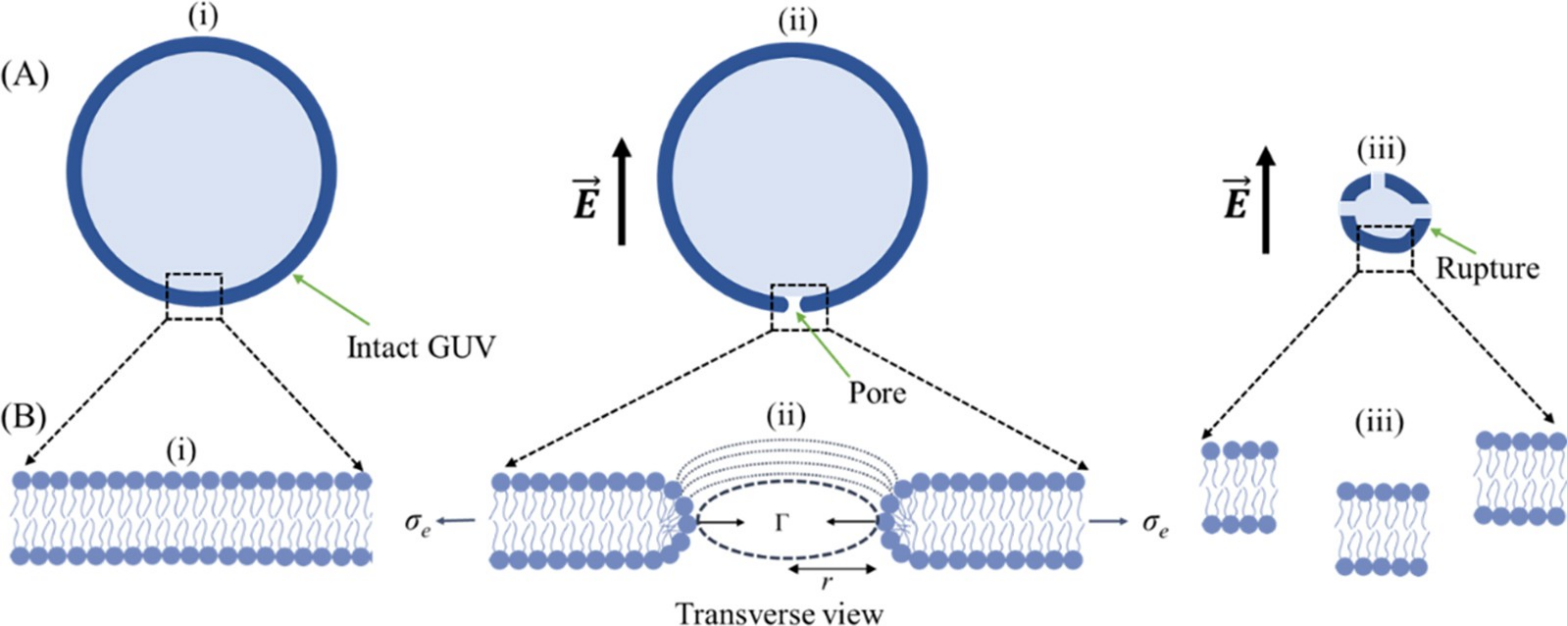
Schematic illustration of the possible steps for the rupture of a ‘single GUV’. (A) A ‘single GUV’ with (i) intact (ii) single pore, and (iii) ruptured conditions. (B) Bilayers with (i) intact (ii) transverse view of a hydrophilic toroidal pore, and (iii) segregated conditions. In (ii) *σ*_e_ is the lateral tension, Γ is the pore edge tension, and *r* is the pore radius. The electric field (*E*) direction is shown with an arrow.

We have conducted the experiments on vesicle rupture using different frequencies. It shows two notable phenomena: vesicle rupture started without alterations in size (Fig 2) and vesicle rupture subsequent to changes in size (Fig 3). A stochastic rupture is observed at each frequency (Fig 4). A rise in frequency leads to a higher occurrence of ruptures (Figs 7 and 8A). The free energy of a prepore in the lipid vesicle is expressed as follows [15, 46]:

$$U(r,\sigma_{e})=2\pi r\Gamma -\pi (\sigma_{e}+B)r^{2}$$ (5)

where Γ represents the free energy per unit length of a prepore (i.e., pore edge tension or line tension), *σ*_e_ is the lateral electric tension, *r* is the radius of a prepore, and the parameter *B* (= 1.76 mNm^-1^) represents the electrostatic interaction arising from the surface charge of membrane [48]. The term -π*r*^2^*σ*_e_ favors prepore expansion and the term 2π*r*Γ favors prepore closure. We specifically considered the toroidal structure of the prepore as illustrated in Fig 10.

The energy barrier for pore formation at critical radius *r*_c_ = Γ/ (*σ*_e_ + *B*) is expressed [49]:

$$U_{b}=\frac{\pi \Gamma^{2}}{\sigma_{e}+B}$$ (6)

According to the Arrhenius equation the rate constant is expressed as follows [50]:

$$k_{p}=A\exp(-\frac{U_{b}}{k_{B}T})$$ (7)

where, *A* is a constant (in s^-1^), *k*_B_ is the Boltzmann constant, and *T* is absolute temperature.

The equivalent circuit of lipid bilayers takes the form of a capacitor (capacitance *C*_m_) in parallel with a resistor (*R*_m_) as shown in Fig 1(D). Basically, *R*_m_ comes from combination of an extracellular resistance (*R*_e_) in parallel with the series combination of the membrane capacitance and an intracellular resistance (*R*_i_). The total impedance of the circuit shown in Fig 1(D) [51]:

$Z=R_{m}X_{c}/(R_{m}+X_{c})=1/(1/R_{m}-j2\pi fC_{m})$, where *R*_m_ is frequency (*f*)-independent and the capacitive reactance *X*_c_ (= −1/*j*2π*f C*_m_) depends on *f*, where *j* indicates the imaginary part. The amplitude of the impedance of the lipid bilayers,

$$|Z|=\frac{1}{\sqrt{\left(1/R_{m}^{2}+4\pi^{2}f^{2}C_{m}^{2}\right)}}$$ (8)

With the increase of frequency of DC pulses, the value of *Z* decreases (i.e., conductance increases). It is to be noted that electroporation intensity can be increased by increasing the pulse amplitude, pulse width (i.e., frequency) and/or pulse number. In our investigations, we changed the frequency of the signal while keeping the membrane tension constant.

The time evolution of pores (*n*) of radius, *r*, is represented as a diffusion process in the potential function Δ*W*_p_ (Δ*W*_p_ has the same physical concept of *U*(*r*, *σ*_e_)), which can be expressed by the Einstein–Smoluchowski equation [51]:

$$\frac{\partial n}{\partial t}=D\frac{\partial }{\partial r}(\frac{\partial n}{\partial r}+\frac{n}{k_{B}T}\frac{\partial \Delta W_{p}}{\partial r})+S$$ (9)

where, *D* is the diffusion coefficient of pores in the space of pore radius, and the source term *S* accounts for the rate of pore creation and annihilation. The parameter Δ*W*_p_ governs the evolution of the pore population, representing the energy required for pore formation in the membrane and can be considered equivalent to the free energy associated with pore formation. The increase in pore formation enhances the conductance in the lipid bilayer, which further supports the discussion mentioned above based on the parallel *R*_m_*C*_m_ circuit. The increase in conductance effectively decreases the energy barrier (i.e., *U*_b_) for electroporation (i.e., rupture) and hence increase the rate constant of rupture according to Eq (7).

Again, the probability of rupture *P*_p_rup_ within 60 s duration of observation is presented as follows [25]:

$$P_{p\_\mathrm{rup}}=\int_{0}^{60}\frac{d(1-P_{\mathrm{int}})}{dt}dt=\int_{0}^{60}k_{p\_\mathrm{rup}}\exp(-k_{p\_\mathrm{rup}}t)dt=1-\exp(-60k_{p\_\mathrm{rup}})$$ (10)

As described earlier in the possible mechanism of vesicle rupture induced by DC pulses of various frequencies (see 4.1), this size reduction of the GUVs can be attributed to the expulsion of lipid molecules from the membrane in the form of small vesicles and/or tubules [40]. The quantity of ejected lipids is directly proportional to the permeabilized membrane area. A microscopy study confirmed a similar lipid ejection phenomenon, thereby affirming that lipid loss is not an artifact of membrane labeling [41]. The observed size change of GUVs at 1250 Hz before rupture (Fig 3) can be elucidated based on the above discussion. Hence, the increase in membrane conductance and the possibility of pore formation at higher frequency results in the higher probability and rate constant of rupture, as obtained in our experiments (see Figs 2, 3, 4, 7 and 8A).

Recently, the deformation of GUVs has been explored at different electric field [1, 52]. The authors introduced a slightly different conductivity between the inside and outside of the GUVs. In addition, they utilized different lipids compared to our study. Thus, they observed a clear change in shape in response to the electric field. In contrast, our research focused on the investigation of GUV rupture and fusion, along with an examination of their kinetics under various frequencies of DC pulses. We maintained identical conductivity both inside and outside the GUVs. It is worthy to mention that the electroporation models are sometimes unable or at least show limitations to explain the experimental data reported recently [53]. Hence the inferences made from the electroporation models should be taken with some care.

Recently, a Boundary Integral Method (BIM) was employed to study an elliptical vesicle placed in a uniform electric field [38]. The investigation primarily focused on the deformation of non-spherical shaped vesicles in the presence of DC electric pulses with the aim of understanding cell electroporation. The computational methodology did not allow for the simulation of actual pore formation due to topological changes in the membrane; rather, it examined tension evolution and distribution in the membranes. Simulation of vesicle dynamics in DC pulses revealed the critical role played by the conductivity ratio, Λ, and field strength in shape evolution. While a vesicle with Λ > 1 remained prolate throughout, with the highest tension at the poles (and hence most likely to porate there), a vesicle undergoing prolate-oblate transition (Λ < 1 and sufficiently high field strength) exhibited more complex tension evolution and was likely to porate in a region between the pole and the equator. These results underscored the importance of shape changes of non-spherical (i.e., elliptical) vesicles during the electroporation process.

In our study, we investigated electroporation (i.e., rupture) and electrofusion of spherical vesicles with a conductivity ratio of Λ = 1. Additionally, the electric field applied to the vesicles was relatively low, ranging from 320 to 340 V/cm. As for simplicity, we considered uniform tension in the membrane induced by electric stress.

Commonly, the pure lipid membrane is considered as an insulator separating two electrically conductive compartments. The equivalent circuit of lipid bilayers takes the form of a capacitor in parallel with a resistor, see Fig 1A.

### 4.2 Electrofusion mechanism

In this experiment, we observed that electrofusion is more pronounced at lower frequencies and decreases at higher frequencies (Figs 5–8B). At lower frequencies, the presence of pores in the contact region of the GUVs promotes their electrofusion. However, at higher frequencies, electrofusion decreases because the GUVs tend to rupture rather than fuse. The precise molecular mechanisms underlying how membrane electroporation facilitates fusion are not fully understood. Previously, a model was proposed suggesting that the electric field induces pores that span across both adjacent membranes in the contact zone [44]. Essentially, the formation of a pore in one of the bilayers could locally increase the electric field, promoting the nucleation of another pore in the adjacent bilayer. If numerous such double-membrane pores are nucleated, they could coalesce into larger loop-like and tongue-like cracks. High-speed optical imaging with a time resolution of ~ 50 μs during the electrofusion process between two GUVs revealed that several double-membrane pores (fusion necks) typically form in the contact zone during the pulse [54, 55]. The expansion and subsequent coalescence of these fusion necks result in the formation of small contact-zone vesicles, which remain trapped within the interior of the fused GUV. This same mechanism is also applicable to the electrofusion of multiple GUVs (Fig 9).

**Fig 11:**
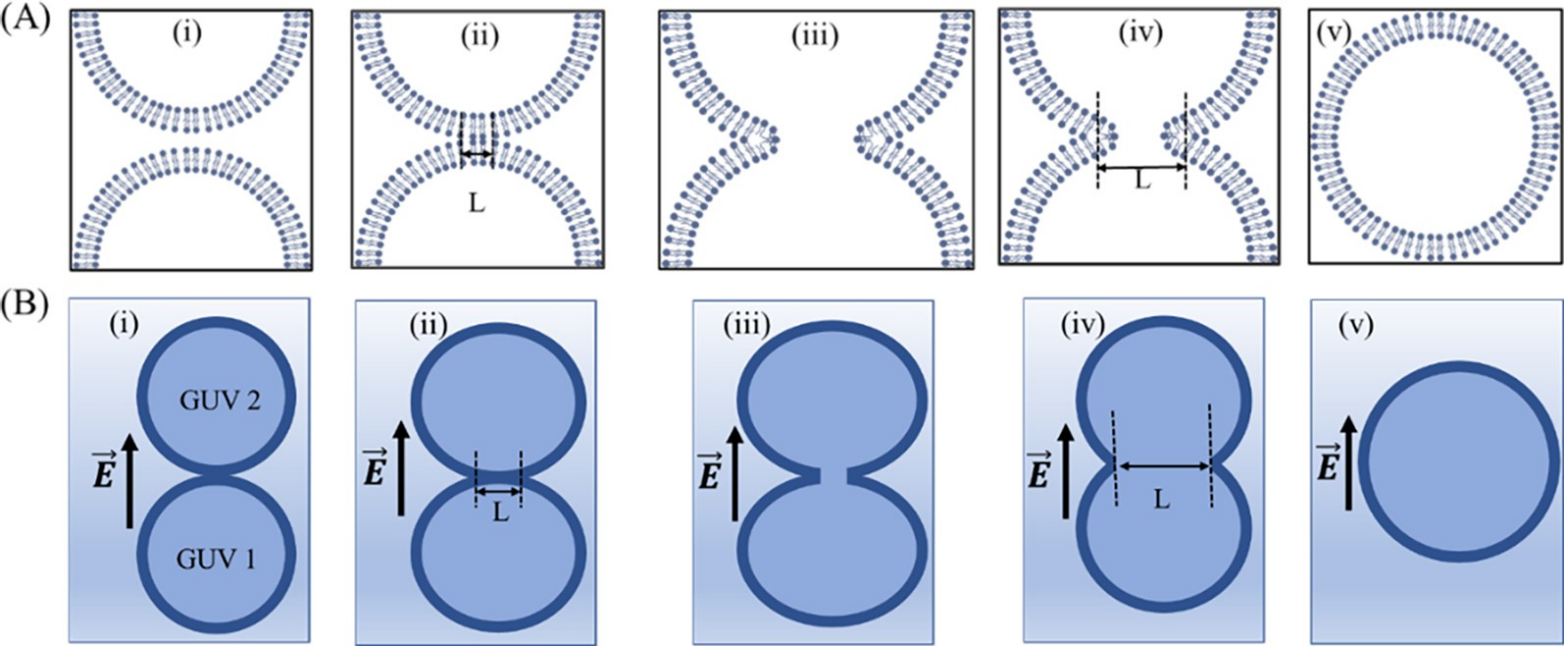
Schematic illustration of the possible steps of ‘stalk mechanism’ of the electrofusion of two ‘single GUVs’. (A) Stepwise changing of (i) two closely approached bilayers (ii) form a stalk following close apposition of the interfaces (iii) the monolayers undergo a continuous deformation toward a trans-monolayer contact (TMS), (iv) the strongly curved dimple ruptures under stress to form a fusion pore, and (v) merged completely. (B) Stepwise changing of two ‘single GUVs’ which are (i) approaching closely, (ii) at contact position, (iii) merged slightly, (iv) merged greatly, and (v) fused completely. The length of fusion neck (L) progresses with time. The electric field (*E*) direction is shown with an arrow.

The most accepted model of electrofusion of lipid vesicles is the ‘The stalk mechanism’ [56]. A schematic illustration of several steps of the electrofusion of stalk mechanism is depicted in Fig 11.

In this mechanism, the membrane fusion is thought to occur through intermediate structure known as ‘stalk’. This stalk is form due to the bending energy (related to bending modulus) of the lipid bilayer [57]. Theoretical analysis of the stalk has allowed to determine the elastic energy associated with this structure and make predictions regarding the possible evolution of the overall process by assuming that the stalk has a shape of revolution of a circular arc. The energy of the stalk is expressed as follows:

$$W_{s}=\pi \kappa [\int_{\mathrm{stalk}}dA{\left(c_{m}+c_{p}-c_{0}\right)}^{2}-\int_{\mathrm{stalk}}dA{\left(c_{\mathrm{init}}-c_{0}\right)}^{2}]$$ (11)

where, *κ* is bending rigidity, *c*_m_ and *c*_p_ are principal curvatures along the meridian and parallel to the body of revolution representing the stalk, and *c*_0_ is the spontaneous curvature. The first integral represents the bending energy of the stalk membrane and the second integral is equal to the bending energy of the initial membrane. Later the stalk structure was revisited [58] by considering the correct shape of the stalk. In the revised form, the stalk is considered completely stress free and the calculated energy become negative instead of high positive value, facilitating the whole fusion process.

In electrofusion experiments, vesicles or cells are typically brought into contact through the application of a low-intensity AC electric field. When the frequency and intensity of the AC field are appropriately chosen, attractive electrostatic interaction forces come into play between individual GUVs, causing them to align linearly in response to the direction of the applied electric field [59]. In our investigations, we found a high population of GUVs during the synthesis process, resulting in many vesicles being interconnected. This outcome was achieved by drying lipids in 3–4 vials and subsequently purifying them together.

## 5. Conclusions

The cell mimetic vesicle rupture and electrofusion were studied under different frequencies of DC pulses, revealing interesting findings. Both the rupture and electrofusion followed stochastic phenomena for many GUVs. Understanding stochastic phenomena is crucial in various scientific disciplines as it allows researchers to make probabilistic predictions. The probability and the kinetics of rupture increase as the frequency increases. The frequency dependent probability and kinetics of electrofusion show the opposite trend. The investigation enables to identify the optimal frequency for effective electroporation or facilitation of electrofusion. These findings can inspire to develop novel liposome approaches for practical biomedical applications.

## Supporting information

S1 File (DOCX)
