# Supplementary material for: Probability and kinetics of rupture and electrofusion in giant unilamellar vesicles under various frequencies of direct current pulses
Source: PLoS One. 2024 Jun 10;19(6):e0304345. doi: 10.1371/journal.pone.0304345 (PMC11164401; doi:10.1371/journal.pone.0304345)
Supplement: S1 File — (DOCX) [file pone.0304345.s001.docx]

**Supporting Information (S)**

**Probability and kinetics of rupture and electrofusion in giant unilamellar vesicles under various frequencies of direct current pulses**

Md. Tariqul Islam Bhuiyan, Mohammad Abu Sayem Karal, Urbi Shyamolima Orchi, Nazia Ahmed, Md. Moniruzzaman, Md. Kabir Ahamed, and Md. Masum Billah

**S1 Investigation of a ‘single GUV’ in the microchamber**

A ‘single GUV’ specifically denotes the individual vesicle selected for investigation and monitoring upon the application of an external electric field. The GUVs were observed using an inverted phase-contrast microscope. S1 Fig. provides a representative field of view depicting several GUVs within a microchamber. In the initial image of the figure (time *t* = 0), no electric field was applied to the GUVs. Despite the presence of multiple GUVs, our focus remained on a single targeted vesicle, observing its dynamics over time. It is noteworthy that whether the untargeted GUVs were ruptured or not was immaterial to our analysis. The targeted GUV ruptured at 39 s and subsequently disappeared in the presence of electric field. It is important to highlight that only one targeted GUV was investigated per microchamber for a maximum period of 60 s. This procedure was replicated in subsequent microchambers. Previously, we followed the same process for investigating the rupture of GUVs under various physiological conditions [1]. The similar protocols were followed for the fusion experiments for several cases.


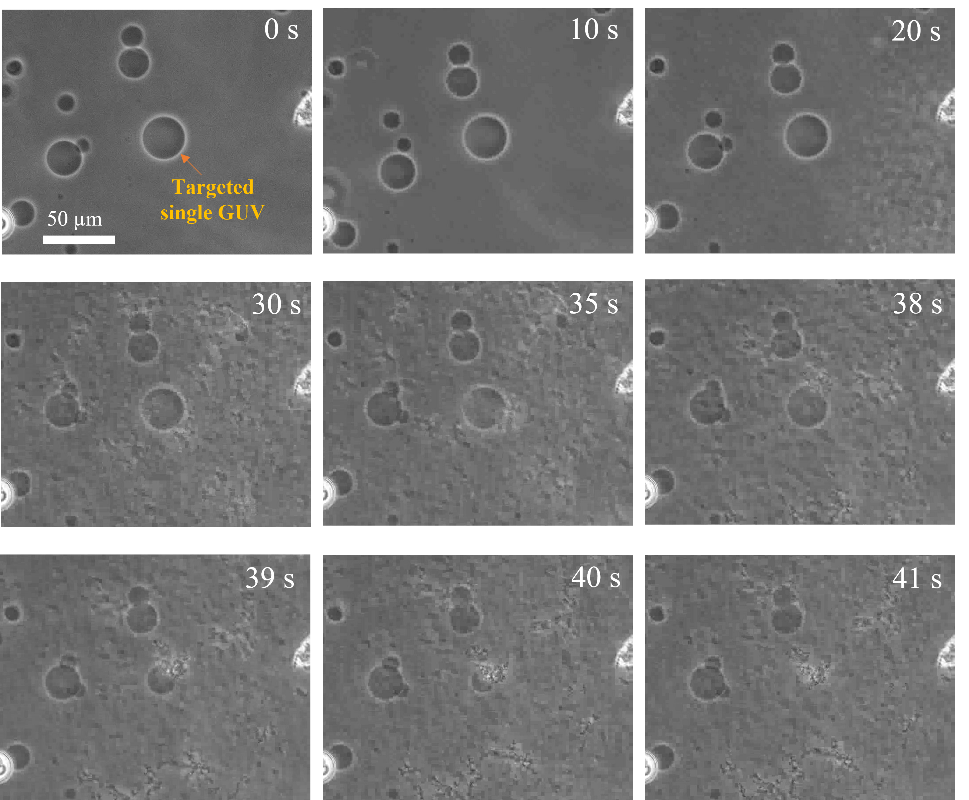


**S1 Fig. Electric field applied on a ‘single GUV’.** The phase contrast images of a ‘single GUV’ at different times.

**S2 Statistical analysis of the probability and rate constant of rupture and fusion using the ‘single GUV’ method.**

The ‘single GUV’ method [2] as illustrated in S2 Fig. was employed for the statistical analysis of the probability and kinetics of rupture and electrofusion. Initially, an electric tension (*σ*_e_) at a specific frequency was applied to a ‘single GUV’, and its structural changes were observed over time (S2A Fig.). Subsequently, the same experiment and observations were conducted for another ‘single GUV’ in the second microchamber under identical conditions. This process was repeated for many ‘single GUV’ (S2B Fig.) in different microchambers. The probability of rupture of all examined GUVs is expressed as, *P*_rup_ = *N*_rup_/*N*_tot_, where *N*_rup_ is the number of ruptured GUVs, *N*_tot_ is the total number of examined GUVs. The time course of the fraction of intact GUVs, *P*_intact_ (*t*), out of all the examined GUVs was fitted with a single exponential decay function (S2C Fig.). From the fitted curve, the rate constant of rupture (*k*_p-rup_) of GUVs was obtained. The rate constant of rupture provided the rate of transition from the intact vesicles to the ruptured vesicles (S2D Fig.). The average rate constant of rupture with standard deviation was calculated under the same condition from several independent experiments. The similar procedure was followed for obtaining the rate constant of electrofusion. The probability of electrofusion of all examined GUVs is defined as, *P*_fus_ = *N*_fus_/*N*_tot_, where *N*_fus_ is the number of fused GUVs and *N*_tot_ is the total number of examined GUVs. The average probability with standard deviation was calculated under the same condition from several independent experiments.


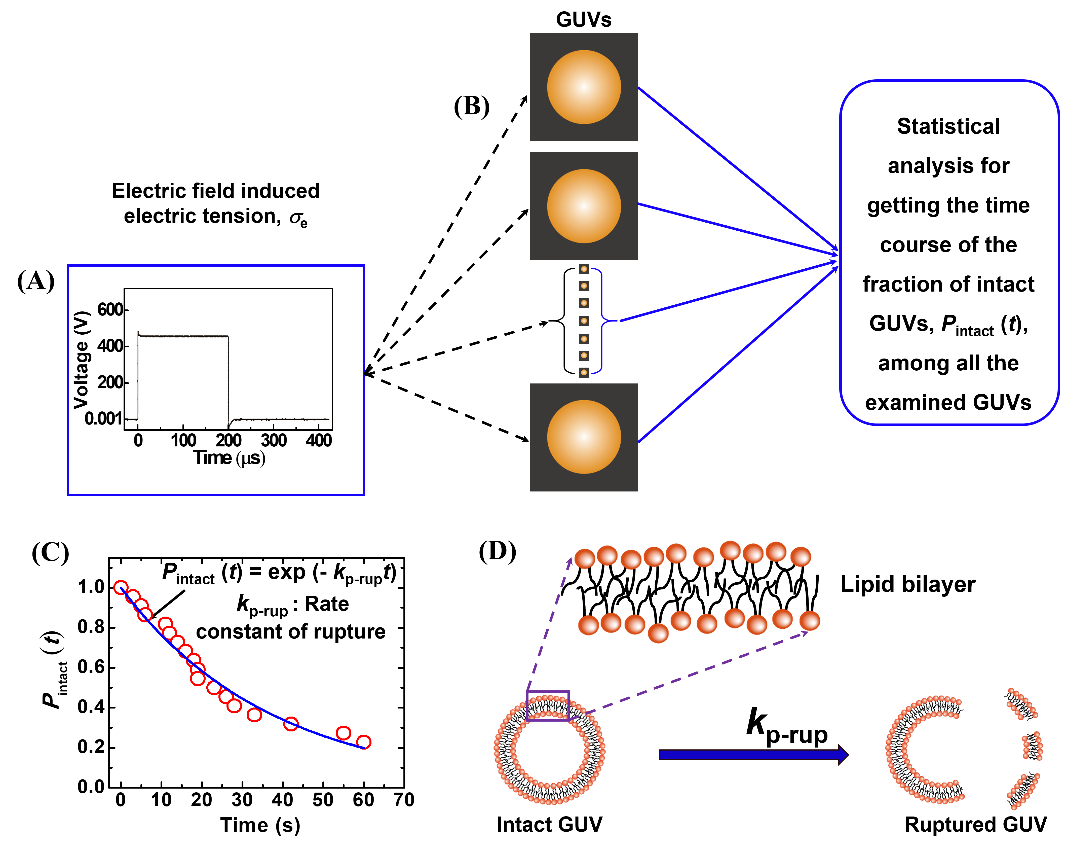


**S2 Fig. Illustration of the ‘single GUV’ method.** (A) Signal of an electric field. (B) Electric field is applied to several ‘single GUVs’. (C) Determination of the rate constant of rupture. (D) Transition from the intact GUVs to the ruptured GUVs.

**References**

1. Karal MAS, Ahamed MK, Ahmed M, Mahbub ZB. Recent developments in the kinetics of ruptures of giant vesicles under constant tension. RSC Adv. 2021;11: 29598–29619. doi:10.1039/D1RA04647K

2. Yamazaki M. The single GUV method to reveal elementary processes of leakage of internal contents from liposomes induced by antimicrobial substances. Advances in Planar Lipid Bilayers and Liposomes. Academic Press; 2008. pp. 121–142. doi:10.1016/S1554-4516(08)00005-7
